# Cost-effectiveness and cost-utility of a Web-based or print-delivered tailored intervention to promote physical activity among adults aged over fifty: an economic evaluation of the Active Plus intervention

**DOI:** 10.1186/s12966-014-0122-z

**Published:** 2014-09-28

**Authors:** Rianne HJ Golsteijn, Denise A Peels, Silvia MAA Evers, Catherine Bolman, Aart N Mudde, Hein de Vries, Lilian Lechner

**Affiliations:** Department of Psychology and Educational Sciences, Open University of the Netherlands, Heerlen, PO Box 2960, 6401 DL Heerlen, The Netherlands; Caphri School of Public Health and Primary Care, Maastricht University, Maastricht, The Netherlands; Department of Health Services Research, Maastricht University, Maastricht, The Netherlands; Trimbos Institute, Netherlands Institute of Mental Health and Addiction, Utrecht, The Netherlands; Department of Health Promotion, Maastricht University, Maastricht, The Netherlands

**Keywords:** Tailored intervention, Older adults, Physical activity, Cost-effectiveness, Cost-utility, Quality of life

## Abstract

**Background:**

The adverse health effects of insufficient physical activity (PA) result in high costs to society. The economic burden of insufficient PA, which increases in our aging population, stresses the urgency for cost-effective interventions to promote PA among older adults. The current study provides insight in the cost-effectiveness and cost-utility of different versions of a tailored PA intervention (Active Plus) among adults aged over fifty.

**Methods:**

The intervention conditions (i.e. print-delivered basic (PB; N = 439), print-delivered environmental (PE; N = 435), Web-based basic (WB; N = 423), Web-based environmental (WE; N = 432)) and a waiting-list control group were studied in a clustered randomized controlled trial. Intervention costs were registered during the trial. Health care costs, participant costs and productivity losses were identified and compared with the intervention effects on PA (in MET-hours per week) and quality-adjusted life years (QALYs) 12 months after the start of the intervention. Cost-effectiveness ratios (ICERs) and cost-utility ratios (ICURs) were calculated per intervention condition. Non-parametric bootstrapping techniques and sensitivity analyses were performed to account for uncertainty.

**Results:**

As a whole (i.e. the four intervention conditions together) the Active Plus intervention was found to be cost-effective. The PB-intervention (ICER = €-55/MET-hour), PE-intervention (ICER = €-94/MET-hour) and the WE-intervention (ICER = €-139/MET-hour) all resulted in higher effects on PA and lower societal costs than the control group. With regard to QALYs, the PB-intervention (ICUR = €38,120/QALY), the PE-intervention (ICUR = €405,892/QALY) and the WE-intervention (ICUR = €-47,293/QALY) were found to be cost-effective when considering a willingness-to-pay threshold of €20,000/QALY. In most cases PE had the highest probability to be cost-effective.

**Conclusions:**

The Active Plus intervention was found to be a cost-effective manner to increase PA in a population aged over fifty when compared to no-intervention. The tailored Active Plus intervention delivered through printed material and with additional environmental information (PE) turned out to be the most cost-effective intervention condition as confirmed by the different sensitivity analyses. By increasing PA at relatively low costs, the Active Plus intervention can contribute to a better public health.

**Trial registration:**

Dutch Trial Register: NTR2297

**Electronic supplementary material:**

The online version of this article (doi:10.1186/s12966-014-0122-z) contains supplementary material, which is available to authorized users.

## Background

Lack of physical activity (PA) is a problem in many developed countries, as almost half of the population does not meet the recommended PA guideline (i.e. being physically active at least 5 days a week, 30 minutes a day with moderate to vigorous intensity) [1,2]. Insufficient PA is a major risk factor for a number of chronic diseases, such as coronary heart disease, stroke, cancer and type 2 diabetes [3]. The World Health Organization estimated that about 3.5% of the total disease burden and 10% of deaths in Europe can be attributed to a lack of PA [4]. The adverse health effects of insufficient PA result in high costs to society in the form of health care costs, productivity losses and costs associated with premature death [3]. Although it is difficult to determine the total cost of insufficient PA on society, as only a fraction of the costs can be estimated [4,5], some estimations have been made. Oldridge [6] mentions that a lack of PA contributes to between 1.5% and 3.0% of direct health care costs in developed countries. Colman and Walker [7] estimated that in a population of 10 million people, where half of the population is too inactive to enjoy health benefits from PA, the costs of insufficient PA can be up to €910 million a year [7]. This burden to society emphasizes the importance of stimulating people to become more physically active, which can result in better public health and thereby reduce health care costs [5,8,9].

Sufficient PA is particularly important for older adults as it enables them to maintain their mobility and independence, to improve their muscle strength, cognitive functioning and mental and emotional well-being, and to prevent falls and chronic diseases [10,11]; additional benefits can be achieved if those who are already physically active further increase their PA [12]. The expectation that the proportion of elderly in Western countries increases (e.g., from 15% in 2010 to nearly 26% in 2040 in the Netherlands [13]), stresses the urgency for cost-effective interventions aimed at promoting PA for older adults. Research has shown that improvements in PA result in savings in health care costs, even within a year [14].

Computer-tailoring has proven to be an effective intervention strategy for promoting PA behavior [15-19]. It is a potentially cost-effective strategy, as it provides the opportunity to give an individual advice to large populations with minimal costs. Several studies have shown that interventions aimed at personal characteristics of participants and interventions using behavioral change strategies (as applied in most tailoring interventions) are most effective in stimulating PA [16]. As a consequence, a computer-tailored intervention to stimulate PA among adults aged over fifty, the Active Plus intervention, was developed and evaluated on effectiveness. This theory-driven, evidence-based intervention is available in four conditions: (1) a basic print-delivered condition (targeting socio-cognitive determinants of PA); (2) an environmental print-delivered intervention (targeting environmental determinants in addition to the basic intervention); (3) a basic Web-based condition; and (4) an environmental Web-based condition [20,21]. The different intervention conditions resulted in different effects on long-term PA behavior; the printed conditions resulted in higher effects than the Web-based conditions [17]. Intervention costs are expected to differ between the different intervention conditions as well; the Web-based conditions are expected to have lower intervention costs than the printed intervention conditions, and additional intervention costs can be expected as a result of providing environmental information. Cost-effectiveness analyses may inform us of which intervention condition effects and costs are optimal.

Until now, in general but also specifically for older adults, very little research has been done to compare the cost-effectiveness of internet-based computer-tailored PA interventions with other PA interventions [2,22,23]. One study by Lewis et al. [24] has shown that an internet-based intervention was cheaper than a print-delivered intervention, however this study did not provide insight in the effects of those interventions. Two other studies comparing print-delivered computer-tailored interventions with phone-delivered computer tailored interventions [18,25], found the print-delivered interventions to be more cost-effective. However, none of these three studies adopted a societal perspective, which is needed to get a complete description of costs and benefits [26]. The Dutch guidelines for economic evaluations also recommend a societal perspective [27]. A societal perspective is the broadest possible perspective, and includes all relevant costs and effects to society (i.e., intervention costs, health care costs, participant and family costs and productivity losses). The absence of this perspective hampers the interpretation of the results of the studies for policy goals.

The purpose of the current study is to evaluate the four Active Plus intervention conditions in terms of costs and effects from a societal perspective. In addition to reporting effects on PA behavior, the results are also presented in terms of Quality Adjusted Life Years (QALYs), enabling policy makers to prioritise between different kinds of interventions in different areas (e.g. comparing the cost-effectiveness of a smoking cessation intervention with a PA intervention).

## Methods

This study is approved by the Medical Ethics Committee of Atrium – Orbis – Zuyd (10-N-36) and was registered in the Dutch Trial Register (NTR 2297).

### Study design and participants

**Figure 1:**
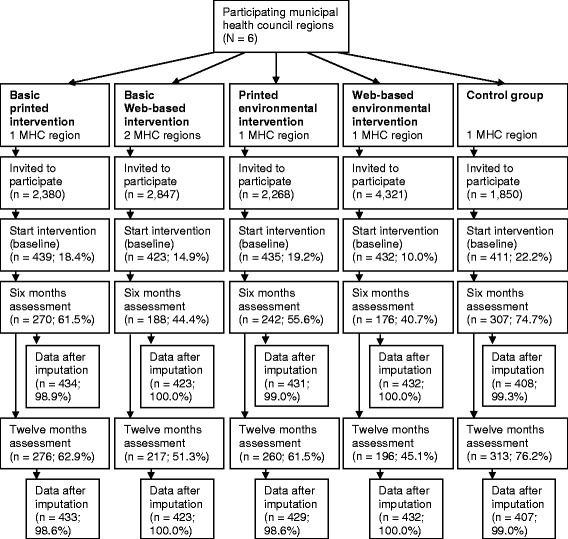
**Participant flow through the Active Plus intervention Note: Percentages of the 6 and 12 months assessment are reported in contrast to the number of baseline participants.**

The four Active Plus intervention conditions (i.e. printed basic (PB), printed environmental (PE), Web-based basic (WB), Web-based environmental (WE)) and a waiting-list control group (C) were studied in a clustered randomised controlled trial. In order to prevent subjects from different intervention conditions contaminating each other, the intervention conditions were randomly assigned to different but comparable municipal health council (MHC) regions. After randomisation, for each intervention condition 14 (matched) neighbourhoods within the MHC region were selected. The neighbourhoods were matched on their urban character (i.e. the number of addresses per km^2^), percentage of people with a low SES, percentage of people with a high SES, and the percentage of people over 50 years of age. A sample of eligible participants from each MHC region received an invitation to participate in the study. For the print-delivered conditions (PB *N* = 2,380; PE *N* = 2,268) this invitation contained an information letter, a questionnaire, a prepaid return envelope, and an informed consent form; the invitation for the Web-based conditions contained an information letter, additional information about how to complete the online questionnaires, a hyperlink to the Active Plus website, and a personal username and password to log in to the website. To reach equal participation rates in the Web-based conditions, a larger sample received an invitation to participate (WB *N* = 2,847; WE *N* = 4,321). For the waiting-list control group (*N = 1,850*) the invitation contained an information letter, a questionnaire, a prepaid return envelope, and an informed consent form. The information letter told the participant that they were invited to complete 4 questionnaires about PA during the upcoming year and that they would receive a PA advice after one year as a reward for their cooperation. The participant flow through the study is graphically depicted in Figure 1. More detailed information about the recruitment of participants and the power calculation can be found elsewhere [28].

There were four evaluation assessments: (1) at the start (T0: also providing data for the first and second tailored advice); (2) three months after baseline (T1: also providing data for the third tailored advice); (3) six months after baseline (T2); and (4) twelve months after baseline (T3). Questions for the economic evaluation were included at baseline, six-, and twelve months.

### Intervention

The Active Plus intervention is a computer-tailored, theory and evidence-based intervention to stimulate or maintain PA among people aged over fifty [20]. During the intervention, participants received a tailored PA advice at three moments based on their personal characteristics, motivational readiness for behaviour change, and needs assessed by previous questionnaires. Psychosocial determinants such as awareness, attitude, self-efficacy, motivation, action planning and coping planning were addressed in the tailored advice. Participants received their first advice within two weeks after completing the baseline questionnaire. The second advice was provided two months after completing the baseline questionnaire. The third advice was provided within four months after baseline measurement, after completing the second questionnaire, enabling us to provide respondents with ipsative feedback about changes in their PA behaviour and the psychosocial determinants in the previous 4 months. This means that improvements in (determinants of) PA were rewarded and possible relapses were addressed appropriately with additional suggestions to increase PA levels again. The intervention was delivered in a printed and a Web-based condition [21] and in a condition with and a condition without additional environmental components (e.g., walking and cycling routes and PA possibilities and initiatives in participants own neighbourhood and home exercises) [29]. Participants in the printed conditions received their advice by mail, whereas participants in the Web-based conditions received their advice through a website and by email. Each tailored advice contained between five and 11 pages of text and illustrations. The specific content of the intervention has been described extensively elsewhere [30].

### Measurements

#### Identification of costs and effects

An economic evaluation was performed from a societal perspective including all relevant costs to society, therefore intervention costs, health care costs, participant and family costs and productivity losses were identified as relevant costs. *Intervention costs* relevant for implementation of the intervention included invitation costs, printing and postage costs, staffing costs for handling questionnaires (which are part of the intervention), advice and reminders, and gathering environmental information, and hosting costs for the tailoring software and website. Response rates are incorporated in the invitation costs (e.g., to include one participant, an information package has to be send out to at least four potential participants). Intervention costs are specified in more detail in Additional file 1. Costs and time attributable to the research (e.g. printing and postage costs for the research part of the questionnaires) and the development of the intervention were excluded, as these costs will not have to be paid in future implementation.

Based on discussion with experts regarding economic evaluations, previous research and economic evaluation literature [27,31-34], the following *health care costs* were assumed to be relevant: costs related to consultation of a general practitioner, lifestyle coach (i.e., dietician, PA advisor), paramedical care provider (e.g. physiotherapist), mental health care provider, company doctor, social worker, practice nurse, medical specialist, or any remaining health care providers. Furthermore, hospital admission and surgery costs were identified as relevant costs, as well as prescribed and over-the-counter medication and paid homecare. *Participant and family costs* consisted of costs incurred for being physically active (i.e., buying sports products and paying membership fees for sports club or gym), travel costs to health care providers and unpaid homecare. Time costs for participating in the intervention were assumed to occur in leisure time and were expected to be reflected in the quality of life of the participants and therefore, were not expressed in monetary value, according to the Dutch guidelines [27]. *Productivity losses* were considered as relevant costs, since a substantial part of the participants was expected to have a paid job, especially in the younger age group.

Intervention effects were expressed in MET-hours per week and QALYs. MET stands for Metabolic Equivalents and presents the amount of energy expended on PA as a multiple of the energy expended while seated at rest. Combining both the duration (hours) and the intensity (MET rate) of PA results in the measure MET-hours of PA. A QALY is a measure of the additional life expectancy resulting from an intervention, corrected for the quality of that life expectancy. A year in perfect health results in a QALY of 1, whereas death results in a QALY of 0. Some health states are valued as being worse than dead and therefore a QALY score can also be negative [33].

#### Measurement of costs and effects

Cost invested and time needed for the implementation of the intervention were registered in detail during the research. *Health care costs*, *participant and family costs* and *productivity losses* were assessed 3 months retrospectively (for medical specialist care and hospital admission 6 months) with a costing questionnaire. For *health care costs*, participants were asked to indicate, if applicable, which health care provider they attended and how often, how many nights they stayed in the hospital, which surgery they had, which medication they used and if they received any paid home care. To estimate *participant and family costs*, questions were included about the money participants spend to pay for their membership at sport accommodations and which sports equipment they bought and to assess if participants received any unpaid home care. Travel costs to health care providers were based on average travel distances and parking fees as described by Hakkaart – van Roijen et al. [27]. Absence from a paid job due to illness (hours in the preceding 3 months) was assessed to calculate *productivity losses*. Cost questionnaires used in previous research were reviewed and adapted to capture the most important information for the current study [35].

The primary outcome measure for the cost-effectiveness analysis was PA expressed in MET-hours per week, assessed with the validated self-administered Dutch Short Questionnaire to Assess Health Enhancing Physical Activity (SQUASH) which has a reasonable reproducibility (r_spearman_ = 0.58; 95% CI = 0.36–0.74) and relative validity (r_spearman_ = 0.45; 95% CI = 0.17–0.66) [36]. A study of Wagenmakers et al. [37] showed that using the SQUASH in an older population can be considered as a fairly reliable tool as well (r_spearman_ = 0.57) and that the validity (varying between 0.20 and 0.67 when compared with the Actigraph) was comparable to those of other questionnaires.

Quality of life, in terms of QALYs, was assessed using the EuroQol (EQ-5D-3L) [38], as is currently recommended by the National Institute for Health and Clinical Excellence (NICE) as measure for assessing quality of life [39]. The EQ-5D-3L is frequently used in economic evaluations of preventive and Web-based interventions [31,40-42].

#### Valuation of costs and effects

For the valuation of *health care costs* and *patient and family costs,* cost prices of the updated Dutch manual for cost analysis in health care research were used [27]. If available, standardised cost prices were used; if unavailable, real costs or average tariffs were used. According to the guidelines, costs of medications were calculated based on defined daily dosages and included 6% Value Added Tax, prescription charges and claw-back, a lawful discount percentage to be subtracted from medication prices by pharmacists [27,43]. Costs spent on sports membership fees and sports equipment were valued based upon the cost prices specified by the participants. For this category, outliers (i.e., participants indicating that they purchased a swimming pool or soccer stadium) were excluded. Prices for unpaid home care were based on shadow prices for domestic care [27]. Travel costs were calculated based on the number of visits to a health care provider, the average travel distances and cost prices and parking fees as described in the Dutch manual for cost analysis [27]. *Productivity losses* for paid work were valued according to mean salaries (differentiated for men and women) and the friction cost method [27]. For some costs items, valuation was difficult as respondents were not specific in their description thus, making valuation impossible (i.e., just mentioning surgery without any further specification). These costs were not valued, but regarded as intangible costs.

For the effects, *weekly MET-hours of PA* per week were calculated. *QALYs* were calculated based on participants’ answers on the five dimensions of the EuroQol (EQ-5D-3L) [38]: mobility, self-care, daily activities, pain/discomfort and depression/anxiety. Each dimension was rated on three levels: no complaints, some complaints and many complaints. The five dimensions were combined into a health state and subsequently, utility values were calculated using preferences elicited from a general Dutch population [40]. An overall QALY score was calculated by multiplying the duration of a health state by the quality weight for the health status (utility score) using all measurements [33]. This indicates that for a perfect health state within one year, a maximum QALY score of 1.0 can be obtained. Consequently, at 6 months follow-up a maximum QALY score of 0.5 can be obtained.

### Statistical analyses

Annual costs were calculated by adding up the costs from the 6-month measurement and the 12-month measurement. However, health care costs and membership fees for sports facilities were assessed 3 months retrospectively, therefore these costs were extrapolated to a period of 6 months in order to have an equal measurement period. Costs for buying sports equipment were assumed to occur less frequently and therefore costs occurring within 3 months were adopted as costs occurring in 6 months. All cost prices were indexed to 2011 costs using the consumer price index of the Netherlands Central Institute for Statistics [44]. Since the time frame in which costs and effects occurred was relatively short (one year for the individual participant), discounting was not necessary [33].

Missing data in cost outcomes and quality of life outcomes were imputed with longitudinal imputation techniques. Missing data on 6 months follow-up were imputed by linear interpolation (i.e., imputation with participants’ mean on the previous and next measurement), whereas the missing data on 12 months were replaced based on the principle of last observation carried forward (LOCF) [33]. Due to seasonal influences, linear interpolation and LOCF were not possible for the physical activity outcomes. Missing data on 6 months were, if available, imputed with the 12-month outcomes, which is a relatively conservative assumption since the effects after 12 months were substantially less than the effects after 6 months. If the 12-month outcomes were not available, the effect of the control group (from similar age and gender subgroups) was applied. Missing data on 12 months were also imputed with the effect of the control group. This is also a relatively conservative assumption, since these persons may have participated in the full intervention.

One-way analyses of variance (ANOVA) with Tukey’s post hoc tests and Chi-square tests were conducted to assess baseline differences between the four intervention conditions and the control group in demographics, PA and utility. Due to the skewed nature of cost data, baseline costs were compared using non-parametric bootstrapping based on repeated sampling (5,000 times) from the observed data, with 95% confidence intervals (95%CI) in percentiles [32]. Correction for baseline differences has not often been applied in economic evaluation as most techniques for baseline correction do not result in patient level corrections, which are necessary for the application of bootstrap techniques and constructing cost-effectiveness acceptability curves [45]. There are only some experimental techniques available to correct for baseline differences [45], therefore we only corrected for baseline differences in the sensitivity analyses.

Mean effects 12 months after the intervention started were analysed using ANOVA’s with Tukey’s post hoc tests and Chi-square tests. A total of 27 participants were excluded from further analyses due to missing outcome measures or being an outlier according to the guidelines of the SQUASH (i.e., reporting PA levels of more than 6,720 minutes per week) [36]. Cost data were compared with non-parametric bootstrapping as described above. Comparisons were made between the control group and each intervention condition. Both printed interventions were compared to both Web-based interventions (PB vs. WB; PE vs. WE) and both basic interventions were compared to both interventions with additional environmental information (PB vs. PE; WB vs. WE).

Incremental cost-effectiveness ratios (ICER) and incremental cost-utility ratios (ICUR) were calculated by dividing the difference in costs by the difference in effects (PA in MET-hours and in QALYs) between the control group and the intervention group as a whole (i.e., the four intervention conditions together), to assess the cost-effectiveness and cost utility of the Active Plus intervention against usual care. Furthermore, comparisons were made between the separate intervention conditions to gain insight into which intervention condition is preferred from a cost-effectiveness perspective. If an intervention condition results in higher effects with lower costs, the intervention is preferred (dominant); an intervention condition with lower effects against higher costs is not preferred (dominated). In case of higher effects and higher costs, or lower effects and lower costs, the preference for an intervention condition depends on how much society is willing to pay for a certain gain in effect. Currently there is no fixed willingness-to-pay (WTP) threshold in the Netherlands and it can be up to €80,000, depending on the disease burden [46]. As it is expected that the disease burden of insufficient PA is relatively low, we used a maximum WTP of €20,000/QALY, which is often used for preventive interventions [46,47]. For PA outcomes no maximum WTP is yet defined.

To assess uncertainty around the ICERs, cost and effect pairs were bootstrapped (1,000 bootstrap replications with a WTP threshold of €20,000). Results of the bootstrap analyses were graphically depicted in a cost-effectiveness acceptability curve (CEAC) presenting the probability of the Active Plus intervention being cost-effective compared to the control group for a range of WTP threshold values. CEACs were also constructed for the different intervention conditions thus, indicating the probability of the intervention conditions to be more cost-effective than the others for a range of WTP thresholds. Based on the CEACs, cost-effectiveness frontiers (CEAF) can be defined, representing which intervention condition has the highest probability of net monetary benefit (NMB) for a range of WTP threshold values. A NMB can be calculated by valuing the difference in effect against the WTP for that effect (in this study €20,000 as specified above) [33].

To deal with uncertainty of parameter estimates, some sensitivity analyses were performed considering the different intervention conditions. The first sensitivity analysis was performed considering outcomes at 6 months follow-up (instead of 12 months follow-up) to gain insight into the cost-effectiveness shortly after ending the intervention, as this would provide insight into the cost-effectiveness when maintenance of the intervention effects was optimised. A second sensitivity analysis was performed with only the participants that reported both costs and effects, i.e., a complete cases analysis. Third, because analysis of baseline cost data showed statistical cost differences between the intervention groups, costs were corrected using a regression correction using the method described by Van Asselt et al. [45]. Age and sex were applied as covariates in this correction. Fourth, an analysis was performed from a health care perspective, only including health care costs, to gain more insight into the cost-effectiveness for the health care setting. The fifth, sixth and seventh sensitivity analyses considered different outcome measures for PA and quality of life: minutes of moderate to vigorous PA per week, days per week with at least 30 minutes PA and utility values calculated using preferences from a general UK population [48]. Furthermore, to increase power and to make some inferences about the preferred delivery method and the inclusion of environmental information, sensitivity analyses (for MET-hours of PA and QALYs) were performed combining several intervention conditions. For the delivery method, both printed (PB and PE) and both online (WB and WE) conditions were taken together and compared to the control condition. With regard to providing environmental information, both basic (PB and WB) and both environmental (PE and WE) conditions were taken together and compared to the control condition.

## Results

### Baseline characteristics

In total 2,140 participants were recruited into the study (see Figure 1). Baseline characteristics of the study population are shown in Table 1. Significant baseline differences were found for age and days of PA per week. The participants in the control group and PE were significantly older than participants in WB (*p* = .001; *p* = .002) and WE (*p* = .000; *p* = .000); furthermore participants in PB were significantly older than WE-participants (*p* = .001). The participants in the control group (*p* = .000) and PE (*p* = .005) performed significantly fewer days with sufficient PA at baseline than WB-participants. Bootstrap analyses revealed baseline cost differences for health care costs, participant and family costs and societal costs. Participants in the control group had significantly higher health care (95% CI: −788 to −37) and societal costs (95% CI: −1,135 to −117) at baseline compared to PE-participants. The control group and PE-participants had significantly lower participant and family costs at baseline than WB-participants (95% CI: 21 to 261).

**Table 1:** **Baseline characteristics, mean and standard deviation (SD)**

	**C (n=411)**	**PB (n=439)**	**PE (n=435)**	**WB (n=423)**	**WE (n=432)**	***F***	**χ** ^**2**^	***P***
Mean age (years)(SD)	64.2 (9.5)	63.1 (8.7)	64.0 (9.4)	61.8 (7.1)	60.8 (7.5)	12.05		0.00^a^
Gender (% men)	49.9	45.9	45.3	52.3	51.3		10.63	0.22
Education (% low)	50.3	43.5	47.3	46.1	47.8		4.04	0.40
Paid job (%)	42.8	40.1	43.9	36.8	40.2		5.14	0.27
Physical activity								
MET-hours PA/week (SD)	45.4 (40.0)	41.6 (37.7)	41.5 (32.1)	42.9 (38.9)	43.0 (40.7)	0.71		0.58
Minutes MVPA/week (SD)	806.7 (786.8)	741.3 (739.1)	711.7 (646.7)	684.8 (719.9)	733.4 (721.0)	1.63		0.16
Days with sufficient PA (SD)	3.8 (2.1)	4.0 (2.0)	3.9 (2.0)	4.3 (2.1)	4.0 (1.9)	4.95		0.00^b^
Mean utility (SD)	0.873 (0.180)	0.870 (0.166)	0.891 (0.150)	0.880 (0.163)	0.890 (0.158)	1.17		0.32
Mean health care costs (SD)	1012 (3751)	713 (1568)	625 (1532)	733 (1372)	686 (1907)	1.94		0.10^c^
Mean participant and family costs (SD)	272 (624)	352 (922)	274 (602)	405 (1060)	331 (1001)	1.78		0.13^d^
Mean productivity losses (SD)	220 (2547)	196 (1069)	199 (1009)	465 (2758)	309 (1694)	1.63		0.17
Mean societal costs (SD)	1683 (4993)	1254 (2315)	1083 (2001)	1583 (3406)	1290 (2876)	2.41		0.05^c^

NB: all costs are expressed in Euro’s.

^a^Post-hoc analysis revealed significant differences: C > WB; C > WE; PB > WE; PE > WB; PE > WE.

^b^Post-hoc analysis revealed significant differences: C < WB; PE < WB.

^c^Bootstrap analysis revealed significant differences: C > PE.

^d^Bootstrap analysis revealed significant differences: C < WB; PE < WB.

At the 12-month follow-up, outcome measures were available for 1,235 participants (57.7%). After imputation, as described in the methods section, outcome measures were available for 2,128 participants (99.4%) six months after baseline, and for 2,124 participants (99.3%) at 12-month follow-up (see Figure 1).

### Costs and effects

Societal costs after 12 months for the Active Plus intervention as a whole were €2,582 compared to €2,737 for the control group. As can be seen in Table 2, societal costs for the different intervention conditions were lowest for PE with €2,306, followed by €2,448 for PB, €2,516 for WE, €2,737 for the control group and €3,052 for WB. Participants in WB had significantly higher productivity losses than participants in the control group and PB (i.e., the bootstrapped 95% CI did not include zero). No significant differences were found in the other main cost categories. Significant differences in subcategories are marked in Table 2. Intervention costs could not be statistically compared, since there is no variance in these costs within a condition.

**Table 2:** **Costs and effectiveness outcomes of the Active Plus intervention conditions at 12 months follow-up**

		**Mean costs (SD) 12 months**					**95% CI**							
**Cost category**	**Unit cost price** ^**a**^	**C**	**PB**	**PE**	**WB**	**WE**	**PB-C**	**PE-C**	**WB-C**	**WE-C**	**PE-PB**	**WE-WB**	**PB-WB**	**PE-WE**
Intervention costs	-	0	25.77	31.21	15.53	18.83	-	-	-	-	-	-	-	-
Health care costs		1682 (200)	1423 (131)	1320 (162)	1437 (129)	1406 (189)	−739 to 181	−873 to 122	−733 to 201	−813 to 267	−508 to 315	−452 to 434	−372 to 349	−593 to 397
General practitioner	29.02^b^	126 (11)	122 (7)	117 (9)	125 (9)	143 (11)	−29 to 21	−36 to 18	−30 to 25	−13 to 48	−25 to 17	−8 to 47	−25 to 19	−55 to 1
Life style coach	27.98^b^	16 (4)	16 (4)	18 (5)	20 (5)	33 (10)	−12 to 12	−12 to 15	−8 to 18	−2 to 39	−11 to 15	−8 to 35	−17 to 8	−38 to 5
Paramedical care	35.96^b^	298 (36)	285 (34)	191 (23)	281 (32)	233 (32)	−111 to 85	−195 to −27*	−112 to 80	−158 to 28	−175 to −14*	−137 to 40	−87 to 95	−121 to 36
Mental health care	82.92^b^	48 (19)	58 (31)	20 (7)	67 (20)	38 (11)	−53 to 92	−72 to 8	−36 to 72	−55 to 30	−112 to 7	−76 to 14	−73 to 71	−43 to 6
Company doctor	72.48^c^	15 (4)	14 (5)	11 (4)	23 (6)	7 (3)	−13 to 12	−14 to 7	−6 to 23	−18 to 1	−15 to 9	−30 to −3*	−25 to 7	−4 to 14
Social worker	67.37^b^	4 (2)	10 (8)	12 (9)	28 (14)	5 (2)	−5 to 25	−5 to 29	1 to 57*	−5 to 8	−20 to 25	−55 to 1	−52 to 12	−7 to 29
Practice nurse	15.81^b^	5 (1)	4 (2)	10 (4)	12 (3)	10 (2)	−4 to 3	−2 to 15	2 to 15*	0 to 10	−2 to 16	−12 to 5	−17 to −2*	−8 to 11
Medical specialist	74.62^b^	135 (11)	120 (11)	130 (11)	138 (11)	150 (14)	−44 to 15	−35 to 27	−27 to 34	−18 to 50	−21 to 41	−22 to 47	−48 to 13	−57 to 15
Hospital admission	473.65^b^	418 (75)	366 (98)	374 (114)	291 (56)	427 (131)	−285 to 201	−288 to 243	−317 to 52	−258 to 334	−281 to 306	−105 to 444	−128 to 307	−401 to 288
Medication	Varying^d^	346 (56)	271 (29)	329 (84)	340 (70)	308 (98)	−208 to 40	−195 to 201	−177 to 184	−229 to 215	−84 to 258	−256 to 229	−234 to 60	−249 to 266
Paid homecare	35.84^b^	270 (119)	172 (28)	116 (28)	99 (31)	88 (20)	−379 to 93	−428 to 34	−451 to 19	−461 to 2	−134 to 21	−87 to 59	−8 to 156	−39 to 97
Other health care	Varying^e^	24 (14)	5 (2)	5 (2)	24 (13)	6 (2)	−51 to 2	−50 to 2	−39 to 38	−50 to 4	−5 to 5	−49 to 2	−50 to 1	−6 to 4
Participant & family costs		567 (47)	604 (57)	631 (70)	648 (68)	585 (59)	−106 to 187	−99 to 231	−76 to 244	−128 to 169	−152 to 209	−243 to 112	−215 to 120	−134 to 232
Physical activity costs	-	371 (30)	387 (39)	363 (33)	349 (36)	285 (30)	−81 to 111	−94 to 81	−115 to 75	−169 to −1*	−126 to 77	−158 to 27	−69 to 140	−8 to 167
Travel costs	Varying^b^	75 (6)	71 (6)	61 (5)	79 (6)	74 (6)	−19 to 12	−28 to 1	−12 to 21	−18 to 16	−25 to 5	−21 to 13	−24 to 9	−29 to 3
Unpaid homecare	12.80^b^	126 (32)	155 (40)	218 (63)	230 (57)	235 (51)	−69 to 135	−41 to 242	−16 to 236	−9 to 229	−80 to 219	−154 to 146	−218 to 54	−172 to 147
Productivity losses	-	485 (117)	369 (118)	296 (102)	986 (246)	526 (159)	−443 to 214	−498 to 113	12 to 1068*	−334 to 448	−383 to 230	−1047 to 99	−1179 to −124*	−632 to 114
Societal costs	-	2737 (258)	2448 (204)	2306 (223)	3052 (301)	2516 (273)	−936 to 345	−1098 to 235	−451 to 1090	−966 to 521	−737 to 454	−1338 to 256	−1339 to 100	−912 to 444
	**Mean effects (SD) 12 months**													
**Effectiveness outcomes**	**C**		**PB**		**PE**		**WB**		**WE**			***F***	***P***	
Δ MET hours PA/week	−2.2 (29.1)		3.0 (28.2)		2.7 (27.9)		0.7 (27.8)		−0.4 (31.6)			2.39	0.05	
Δ Minutes MVPA/week	−72.1 (500.9)		3.1 (533.8)		37.7 (506.1)		−32.8 (445.2)		−39.8 (522.7)			2.96	0.02	
Δ days with sufficient PA	0.1 (1.7)		0.4 (1.7)		0.5 (1.6)		0.0 (1.4)		0.2 (1.3)			5.87	0.00	
QALY-EQ-5D-3L	0.884 (0.164)		0.876 (0.155)		0.883 (0.160)		0.883 (0.162)		0.888 (0.165)			0.339	0.85	

C = control group, PB = printed basic intervention, PE = printed environmental intervention, WB = Web-based basic intervention, WE = Web-based environmental intervention.

^a^Unit cost prices are indexed to 2011 costs, based on consumer price index [44]; ^b^based on Dutch guidelines [27]; ^c^based on Collective Agreement for company doctors in the Netherlands (2002); ^d^adapted from www.medicijnkosten.nl based on Defined Daily Dose [43]; ^e^adapted from several resources including Hakkaart – van Roijen et al. [27], detailed specification available upon request; *Significant difference (the 95% CI does not include zero).

Regarding the effects, the Active Plus intervention, as a whole, resulted in a significant increase of 1.5 MET-hours of PA per week (*p =* .02), compared to a decrease of 2.2 MET-hours of PA per week in the control group. Considering the different intervention conditions, no significant differences were found for MET-hours of PA per week, although the difference between participants in PB and the control group was borderline significant (*p* = .067).

No significant differences in QALYs were found either in comparing the Active Plus intervention as a whole to the control group, or in comparing the different intervention conditions to each other and the control group (see Table 2).

### Cost-effectiveness analyses

**Figure 2:**
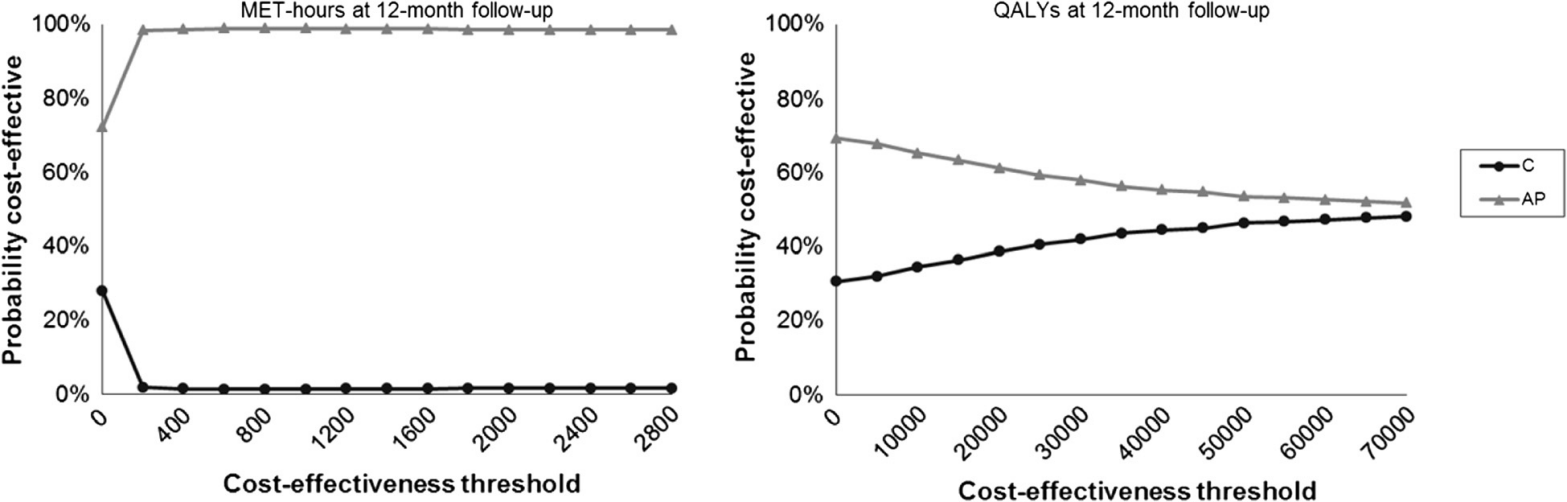
**Cost-effectiveness acceptability curves (CEAC) comparing the intervention as a whole (AP) to the control group (C).**

Comparing the costs and effects of the Active Plus intervention as a whole (*N = 1,692*) to the control group (*N =401*) dominated the control group. Participants in the intervention increased their PA on average 3.8 MET-hours per week with a cost saving of €174 per participant (on a yearly basis) compared to the control group. As can be seen in the CEAC in Figure 2, the Active Plus intervention as a whole was preferred over the control group for all WTP thresholds, with a probability of Active Plus being cost-effective of 72 to 99%.

Considering the costs and effects of the different intervention conditions PB (*N = 428*), PE (*N = 421*) and WE (*N = 425*) all had lower costs and higher effects on MET-hours compared to the control group: all three dominated the control group. ICERs are shown in Table 3. Participants in the PB condition increased their PA with (on average) 5.3 MET-hours/week at a yearly cost saving of €288 per participant. For PE and WE this was an increase of 5.0 MET-hours/week at a yearly cost saving of €464 per participant and an increase of 1.8 MET-hours/week at a yearly cost saving of €255 per participant respectively. The ICER comparing WB (*N = 418*) to the control group indicated that WB had higher costs, but also higher effects than the control group. Participants in this condition increased their PA on average 3.0 MET-hours/week at a yearly cost of €318 per participant compared to the control group. Whether WB dominates the control group depends on the willingness-to-pay for each additional MET-hour of PA, however, as mentioned in the methods, no WTP for MET-hours is yet available.

**Table 3:** **Incremental cost-effectiveness (ICER) and cost-utility (ICUR) ratios of the Active Plus intervention conditions**

	**Cost-effectiveness analysis**			**Cost-utility analysis**		
	**Incremental costs** ^**a,b**^	**Incremental MET-hours/week**	**ICER** ^**c**^	**Incremental costs** ^**a,b**^	**Incremental QALYs** ^**d**^	**ICUR** ^**c**^
***Intervention vs. control***						
Intervention as a whole	−174	3.8	−46^e^	−153	−0.002	101,169^f^
PB	−288	5.3	−55^e^	−315	−0.008	38,120^f^
PE	−464	5.0	−94^e^	−434	−0.001	405,892^f^
WB	318	3.0	108^h^	364	−0.001	440,164^g^
WE	−255	1.8	−139^e^	−211	0.004	−47,293^e^
***Environment vs. basic***						
PE vs. PB	−176	−0.3	555^h^	−119	0.007	−16,516^e^
WE vs. WB	−573	−1.1	514^h^	−575	0.005	−108,851^e^
***Print vs. Web***						
PB vs. WB	−606	2.3	−261^e^	−679	−0.007	91,336^f^
PE vs. WE	−209	3.1	−67^e^	−223	−0.006	40,426^f^

C = control group, PB = printed basic intervention, PE = printed environmental intervention, WB = Web-based basic intervention, WE = Web-based environmental intervention.

^a^In Euros; ^b^differences in incremental costs for the ICER and ICUR occur due to differences in the number of participants for which MET-hours/week and QALYs were available; ^c^calculated according to the formula ICER (or ICUR) = (Costs_i_-Costs_c_)/(Effect_i_-Effect_c_); ^d^Based on the Dutch algorithm for the EuroQol (EQ-5D-3L) scores; ^e^dominant; ^f^dominant based on WTP = €20,000 (i.e. savings larger than WTP); ^g^dominated; ^h^preferred intervention depends on WTP (unknown for PA).

**Figure 3:**
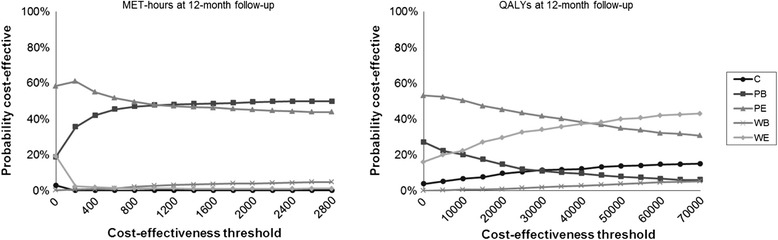
**Cost-effectiveness acceptability curves (CEAC) comparing the different intervention conditions.**

When comparing both environmental interventions to both basic interventions, the environmental interventions have lower costs (PE-PB: €-176; WE-WB €573) than the basic interventions, but at the expense of lower effects (PE-PB: −0.3 MET-hour/week; WE-WB: −1.1 MET-hours/week). The preferred intervention therefore depends on the WTP. Both printed interventions (PB and PE) had lower costs and higher effects at the 12-month follow-up and were thus, dominant over both Web-based interventions (WB and WE). Indicating that participants in the PB condition increased their PA with (on average) 2.3 MET-hours/week at a yearly cost saving of €606 per participant compared to participants in the WB condition. For participants in the PE condition compared to participants in the WE condition this was an increase of 3.1 MET-hours/week at a yearly cost saving of €209 per participant. The CEAC in Figure 3 demonstrates that PE was the preferred intervention condition for WTP thresholds below €1,000 and PB for WTP thresholds higher than €1,000, with a probability of 50% to be the most cost-effective alternative.

### Cost-utility analyses

As a whole, the Active Plus intervention (*N = 1,662*) resulted in lower costs (incremental costs = €-153), but also in fewer QALYs compared to the control group (*N = 402*; incremental effects = −0.002 QALY), resulting in an ICUR of 101,169. Because both incremental costs and effects are negative, it might be difficult to interpret the ICUR. In this context the ICUR indicates how much society would save for a decrease of one QALY, thus the ICUR should be at least €20,000, to have enough savings to compensate for the loss of one QALY. Since the ICUR was higher than the WTP of €20,000 the Active Plus intervention dominated the control group. Figure 2 showed that, as a whole the Active Plus intervention had a probability of 61% being cost-effective at a WTP of €20,000 compared to the control group.

Comparing the different intervention conditions with regard to QALYs indicated that WE (*N = 408*) had higher effects and lower costs and thus dominated the control group. Participants in the WE condition gained 0.004 QALY with a yearly cost saving of €211 per participant compared to the control group. Although PB (*N = 430*) and PE (*N = 418*), had lower costs (PB-C: €-315; PE-C: €-434) at the expense of lower effects (PB-C: −0.008 QALY; PE-C: −0.001 QALY) they still dominated the control group based on a WTP of €20,000 as cost savings could compensate the decrease in QALYs. WB (*N = 406*) had higher costs (€364) and lower effects (−0.001 QALY) and was thus dominated by the control group.

Comparing the environmental interventions to the basic interventions, PE and WE both had higher effects and lower costs and were thus dominant to PB and WB. Participants in the PE condition gained (on average) 0.007 QALY at a yearly cost saving of €315 per participant compared to participants in the PB condition. For participants in the WE condition compared to participants in the WB condition this was a gain of 0.005 QALY at a yearly cost saving of €575 per participant. Both printed interventions, PB and PE, had lower effects (PB-WB: −0.007 QALY; PE-WE: −0.006 QALY) and lower costs (PB-WB: €-679; PE-WE: €-223), but dominated their Web-based counterparts (WB and WE) based on a WTP of €20,000. The ICURS are shown in Table 3. PE had the highest probability to be cost-effective for WTP thresholds below €45,000 as can be seen in Figure 3. At a WTP of €20,000 the probability of PE being cost-effective was 45%.

### Sensitivity analyses

In the sensitivity analyses for the different intervention conditions, PE is predominantly found to be the dominant intervention condition with probabilities of being cost-effective ranging from 38 to 83% (Table 4). In the primary analysis for PA at the 12-month follow-up, PE was also dominant for lower WTP values, whereas PB was the dominant intervention condition for WTP thresholds above €1,000 (Table 4). The sensitivity analysis in which a baseline correction was applied and the analysis from a health care perspective showed the same result as the primary analysis. The complete cases analysis showed an almost similar result although at the lowest WTP thresholds (WTP < €50) WE was the dominant intervention condition. In the sensitivity analysis with MET-hours of PA per week and costs at the 6-month follow-up, PE turned out to be the most cost-effective intervention condition, as was the case in the sensitivity analyses with regard to different PA outcomes. Sensitivity analyses for QALY were similar to the primary analysis for QALY, with PE being the dominant intervention condition.

**Table 4:** **Primary and sensitivity analyses of the Active Plus intervention conditions at the 12-month follow-up**

	**C**		**PB**		**PE**		**WB**		**WE**		**ICER** ^**b**^	**CEAF** ^**d**^ ** and probability highest NMB**
	**Costs** ^**a**^	**Effect**	**Costs** ^**a**^	**Effect**	**Costs** ^**a**^	**Effect**	**Costs** ^**a**^	**Effect**	**Costs** ^**a**^	**Effect**		
**Primary analysis**												
PA (MET-hours/week)	2760 (5301)	-2.2	2472 (4269)	3.0	2295 (4694)	2.7	3078 (6301)	0.7	2504 (5666)	-0.4	555 (PE vs PB)	<€1000 PE, >€1000 PBPB: 50%; PE: 43%
QALY-EQ-5D-3L^c^	2739 (5293)	0.884	2424 (4212)	0.876	2305 (4642)	0.883	3103 (6378)	0.883	2528 (5730)	0.888	40426 (PE vs WE)	<€40000 PE, >€40000 WE; PE: 45%
**Sensitivity analyses**												
*6-month follow-up*												
PA (MET-hours/week)	1444 (2988)	7.4	1329 (2880)	11.5	1106 (2456)	15.6	1501 (3207)	13.9	1201 (2663)	8.1	PE dominant	PE at any threshold; PE: 74%
QALY-EQ-5D-3L^c^	1442 (2985)	0.442	1307 (2860)	0.438	1106 (2426)	0.441	1547 (3305)	0.442	1211 (2683)	0.444	38110 (PE vs WE)	<€45000 PE, >€45000 WE; PE: 56%
*Complete cases*												
PA (MET-hours/week)	2382 (3880)	-2.5	2592 (4666)	6.3	2316 (4986)	5.7	2824 (5241)	1.7	2217 (3316)	4.1	472 (PE vs PB)	<€50 WE, >€50 < €400 PE, >€400 PB; PB: 44%
QALY-EQ-5D-3L^c^	2488 (4212)	0.902	2423 (4452)	0.878	2166 (4668)	0.898	2635 (5031)	0.893	2198 (3774)	0.892	PE dominant	PE at any threshold; PE: 46%
*Baseline correction*												
PA (ME- hours/week)	2862 (3742)	-2.2	2664 (3277)	3.0	2614 (3824)	2.7	3038 (4593)	0.7	2794 (4618)	-0.5	157 (PE vs PB)	<€175 PE, >€175 PB; PB: 54%
QALY-EQ-5D-3L^c^	2851 (3737)	0.884	2633 (3252)	0.876	2622 (3811)	0.883	3045 (4643)	0.883	2795 (4667)	0.888	31308 (PE vs WE)	<€30000 PE, >€30000 WE; PE: 38%
*Health care perspective*												
PA (MET-hours/week)	1693 (4093)	-2.2	1441 (2817)	3.0	1297 (3340)	2.7	1438 (2658)	0.7	1419 (3963)	-0.4	452 (PE vs PB)	<€400 PE, >€400 PB; PB: 53%
QALY-EQ-5D-3L^c^	1670 (4082)	0.884	1386 (2729)	0.876	1287 (3256)	0.883	1428 (2664)	0.883	1386 (3965)	0.888	17966 (PE vs WE)	<€12500 PE, >€12500 WE; WE: 42%
*Other outcome measures*												
Minutes MVPA/week	2749 (5264)	-72.1	2463 (4241)	3.1	2306 (4696)	37.7	3078 (6301)	-32.8	2504 (5666)	-39.8	PE dominant	PE at any threshold; PE: 83%
Days with sufficient PA	2720 (5349)	0.1	2520 (4303)	0.4	2341 (4740)	0.5	3076 (6286)	0.0	2503 (5645)	0.2	PE dominant	PE at any threshold; PE: 76%
QALY-EQ-5D-3L UK tariff	2739 (5293)	0.858	2424 (4212)	0.846	2305 (4642)	0.856	3103 (6378)	0.857	2528 (5730)	0.864	25001 (PE vs WE)	<€25000 PE, >€25000 WE; PE: 42%

PB = printed basic intervention, PE = printed environmental intervention, WB = Web-based basic intervention, WE = Web-based environmental intervention, C = control group.

^a^In Euros; ^b^calculated according to the formula ICER = (Ci-Cc)/ (Ui-Uc); ^c^Based on the Dutch algorithm for the EuroQol (EQ-5D-3L) scores; ^d^Cost-effectiveness acceptability frontiers (CEAF); ^e^Probability of highest net monetary benefit (NMB) based on WTP = €20,000.

Analyses comparing only three groups were done to make some more powerful inferences about preferred delivery mode and the inclusion of environmental information. A printed intervention delivery was found to be the dominant delivery mode in the analyses in which both printed interventions, both Web-based interventions and the control group were compared, with probabilities of 96% (for MET-hours of PA) and 65% (for QALYs), as can be seen in Table 5. Analyses in which the effect of adding environmental were examined revealed that the basic intervention was dominant for MET-hours of PA, whereas the environmental intervention was dominant for QALY outcomes with probabilities of 68% (MET-hours of PA) and 75% (QALYs) (see Table 5).

**Table 5:** **Sensitivity analyses comparing 3 groups of the Active Plus intervention at the 12-month follow-up**

	**Control group**		**Print-delivered intervention (PB & PE)**		**Web-based intervention(WB & WE)**		**ICER** ^**b**^	**CEAF** ^**d**^ **and probability highest NMB**
	**Costs** ^**a**^	**Effect**	**Costs** ^**a**^	**Effect**	**Costs** ^**a**^	**Effect**		
PA (MET hours/week)	2760 (5301)	−2.2	2384 (4483)	2.9	2789 (5992)	0.2	Print dominant	Print at any threshold
								Print: 96%
QALY-EQ-5D-3L^c^	2739 (5293)	0.884	2365 (4427)	0.879	2800 (6054)	0.886	79196 (Print vs. C)	<€65000 Print, >€65000 C
								Print: 65%
	**Control group**		**Basic intervention (PB & WB)**		**Environmental intervention (PE & WE)**		**ICER** ^**b**^	**CEAF** ^**d**^ **and probability highest NMB**
	**Costs** ^**a**^	**Effect**	**Costs** ^**a**^	**Effect**	**Costs** ^**a**^	**Effect**		
PA (MET hours/week)	2760 (5301)	−2.2	2771 (5375)	1.9	2400 (5203)	1.2	503 (Environment vs. Basic)	<€550 Environment, >€550 Basic
								Basic: 68%
QALY-EQ-5D-3L^c^	2739 (5293)	0.884	2754 (5381)	0.879	2415 (5206)	0.886	Environment dominant	Environment at any threshold
								Environment: 75%

PB = printed basic intervention, PE = printed environmental intervention, WB = Web-based basic intervention, WE = Web-based environmental intervention, C = control group.

^a^In Euros; ^b^calculated according to the formula ICER = (Ci-Cc)/(Ui-Uc); ^c^Based on the Dutch algorithm for the EuroQol (EQ-5D-3L) scores; ^d^Cost-effectiveness acceptability frontiers (CEAF); ^e^Probability of highest net monetary benefit (NMB) based on WTP = €20,000.

## Discussion

The purpose of the current study was to compare the Active Plus intervention to a control group in terms of cost-effectiveness (MET-hours) and cost-utility (QALYs). Furthermore, the four intervention conditions (i.e., printed basic, printed environmental, Web-based basic, and Web-based environmental) and the control group were compared to each other. To our knowledge, this is the first study comparing the cost-effectiveness and cost-utility of a computer-tailored physical activity advice, delivered either in a printed fashion or via the Internet. Although a number of studies reported comparisons of Web-based interventions with print-delivered interventions, none of these, published information regarding cost-effectiveness [2,22].

### Cost-effectiveness of the intervention

The Active Plus intervention was cost-effective as it resulted in lower societal costs and an increase in MET-hours of PA per week compared to the control group. With regard to the cost-effectiveness of the different intervention conditions, it was found that both printed interventions (PB and PE), and the Web-based intervention with environmental information (WE) were cost-effective compared to the control group, as all three resulted in lower costs and higher effects. This indicates that the implementation of the intervention in general, and specifically these three intervention conditions requires some upfront investment of money, but even in one year, this already results in cost savings to society (mainly due to decreased health care costs). The basic Web-based intervention (WB) resulted in a higher increase in MET-hours of PA per week compared to the control group, but also in higher societal costs. The cost-effectiveness of the WB intervention condition is thus, dependent on the willingness-to-pay for each additional MET-hour.

Although one might think that a Web-based intervention has the highest potential to be cost-effective compared to a printed intervention, as is also shown in a cost-analysis by Lewis [24], the results from the current study (including a societal perspective, in contrast to Lewis [24]) showed otherwise. Comparing all intervention conditions with each other showed that, although depending on the WTP, both printed interventions had the highest probability of being cost-effective despite the fact that they have the highest intervention costs. The high probability of cost-effectiveness of these intervention conditions might be explained by the fact that these conditions resulted in the highest intervention effects, which might have resulted in savings in health care costs and thereby lower societal costs. Previous research has also shown that changes in PA result in decreased health care charges in the short term. [14] The lower effects of the Web-based intervention conditions can also be explained by the lower use of several intervention components [30] and the higher dropout within the intervention period when compared to the printed intervention [28]. Furthermore, costs from productivity losses (i.e., sick leave) were highest in the Web-based conditions (significantly in WB), resulting in higher societal costs and thus, less positive cost-effectiveness ratios, which might be explained by the fact that different kinds of people respond to the different types of interventions. For example, people participating in the Web-based intervention were significantly younger than participants of the printed intervention. The (younger) Web-based participants were thereby more likely to spend more hours at work than the printed intervention participants, resulting in a higher susceptibility for sick leave, and thus higher potential productivity losses.

As mentioned above, the preferred intervention depends on the WTP. PE had the highest probability of being cost-effective for WTP-thresholds up to €1,000/MET-hour, whereas PB had the highest probability of being cost-effective for thresholds above €1,000/MET-hour. This turning point can be explained by the fact that PB had the highest effects and PE the lowest societal costs, thus, if society is willing to pay more for an additional MET-hour, PB becomes the preferred intervention as it results in higher effects. Determining which intervention condition is most cost-effective is difficult as there is currently no information about how much society is willing to pay for an additional MET-hour, as it is unclear for other health behaviour outcomes [31,41,42]. Therefore, it is desirable if future research is aimed at identifying maximum WTP thresholds for health behaviours.

Several reviews described the cost-effectiveness of PA interventions; however the majority of the included studies were not conducted from a societal perspective [9,49]. The only study conducted from a societal perspective reported an ICER of €825 for one inactive participant to become norm active [50], i.e., an increase of 10 MET-hours [11]. The Active Plus intervention in general and specifically the PB, PE and WE conditions were obviously more cost-effective than the intervention reported by Elley et al. [50], since they would result in cost savings. The costs of the WB condition for getting an inactive participant to become norm active would be (10 times €108/MET-hour) €1,008, which is higher than that reported by Elley et al. [50]. However, the Active Plus study population includes both inactive as well as norm-active participants and is not specifically targeted for an inactive population. A meta-analysis by Woodcock et al. [12] showed that the largest health gains (and thus the largest health care cost savings) occur for the first 15–29 PA-minutes per day by inactive people. This would indicate that if the Active Plus intervention was only implemented in an inactive population, Active Plus would result in even larger health effects, and the ICER would probably be lower than reported in this study, making the WB condition comparably cost-effective to the intervention reported in the study by Elley et al., and the PB, PE and WE condition even more cost-effective than the intervention by Elley et al. [50]. Further research should provide insight in the differences in cost-effectiveness of the intervention in inactive populations when compared to the population in general.

### Cost-utility of the intervention

Assessing cost-effectiveness in terms of QALYs allows for comparing interventions aimed at different health behaviors. Results showed that although, compared to the control group, PA increased more in the Active Plus intervention as a whole, this was not reflected in more QALYs. Nevertheless, the Active Plus intervention had the highest probability of being cost-effective for WTP-thresholds up to €70,000, since it results in savings of societal costs. With regard to the different intervention conditions, PB, PE and WE were found to be cost-effective when considering a WTP of €20,000/QALY. PE had the highest probability (45%) to be cost-effective at a WTP of €20,000/QALY. Although there is a WTP available for QALYs [46,47], making it possible to draw conclusions on cost-effectiveness, differences in QALYs were very small (i.e., the largest observation was an effect of -.008), insignificant and clinically irrelevant, making it difficult to draw conclusions. However, the size of the effect was comparable with other studies with the same time frame [51]; a positive relationship between PA and quality of life is often not found in longitudinal research [52,53]. It is assumed that most health benefits of PA that are reflected in quality of life only become visible when follow-up time increases (beyond the trial period). Since such a long follow-up period is often unfeasible, modeling cost-effectiveness to increase the time horizon is recommended [54]. Furthermore, according to the Set-Point Model, it is assumed that quality of life is quite stable, and only varies temporarily from a certain baseline level in major life events [18,55,56], and according to the Response Shift Theory, participants may have adapted to the new situation [57]. A third possible reason for not detecting substantial differences in QALYs might be the generic measure used in the current study. It is known that the EuroQol is not sensitive enough to detect changes in quality of life in relatively healthy populations such as the participants of the Active Plus intervention [58]. The development of a measure that is more sensitive to changes in a healthy population is necessary, allowing for cost-utility analyses in future economic evaluations of public health interventions [59,60].

### Sensitivity analyses

Although there were some differences from the main analysis with regard to probability of being cost-effective, in most cases PE had the highest probability of being cost-effective at lower WTP values, indicating that results are robust. In the case that intervention maintenance is optimised (first sensitivity analysis), PE still had the highest probability of being cost-effective.

In the primary analysis an imputed dataset was used for increased power. Since fairly similar results were found for the complete case analysis (both for PA and QALYs) it seems that the results are robust for the methods used to impute incomplete data.

Sensitivity analyses were performed with three groups (the control group, both printed interventions together and both Web-based interventions together) to make more powerful inferences about the preferred delivery mode and the addition of environmental information. Results indicated that a printed intervention was preferred. With regard to adding environmental information, results were less clear. For PA outcomes adding environmental information was preferred at lower WTP thresholds, whereas a basic intervention was most cost-effective at higher WTP thresholds. With regard to QALYs an environmental intervention was preferred; this is in line with PE being the most cost-effective intervention condition at the lower WTP thresholds.

### Strengths and limitations

Although the results from this study come from a strong (societal) perspective, the current study has some limitations that should be noticed. One of these limitations is the high number of participants dropping-out of the study and the associated missing values. However, the drop-out rate in the current study is comparable with other studies [22,41,42]. Furthermore, missing values were imputed conservatively (i.e., the PA effect of non-responding participants was assumed to be equal to the control group). This is more conservative than applying multiple imputation, since multiple imputation might overestimate intervention effects [61].

Furthermore, self-reported measurements were used, which can result in measurement bias in the form of social desirable answers and recall bias. However, no differences in measurement or recall bias were expected between the groups. For PA, self-administered questionnaires are the most commonly used, as this is the most inexpensive method to use in large-scale studies. However, validating the intervention effects with an objective measure (e.g., an accelerometer) is recommended.

Despite these limitations, to our current knowledge this is the first study comparing the cost-effectiveness and cost-utility of a computer-tailored physical activity advice, delivered either in a printed fashion or via the Internet. This economic evaluation, in contrast to most other studies [9,49], is carried out from a strong study design, including longitudinal observations and a societal perspective, needed to get a complete description of costs and benefits [26]. Other strengths include the randomised controlled trial [62,63] and the large study population of more than 2,100 participants.

## Conclusion

From the current study it can be concluded that a tailored PA intervention is a cost-effective strategy to promote PA behaviour in adults aged over fifty. Specifically, the printed basic, the printed environmental and the Web-based environmental condition were cost effective tailoring strategies since these resulted in increased PA behaviour and lower societal costs (mainly due to decreased health care costs). Printed and Web-based tailoring interventions to promote PA behaviour in adults aged over fifty can thus, contribute to individual health and thereby also to public health by increasing PA against acceptable costs.

## Additional file

Additional file 1:
**Specification of intervention costs.** This file gives a detailed specification of the intervention costs.

## Abbreviations

95% CI: 95% Confidence interval
ANOVA: Univariate one-way analyses of variance
AP: Active plus
C: Control group
CEAC: Cost-effectiveness acceptability curve
CEAF: Cost-effectiveness acceptability frontiers
ICER: Cost-effectiveness ratio
ICUR: Cost-utility ratio
LOCF: Last observation carried forward
MHC: Municipal ’health council
MVPA: Moderate to vigorous physical activity
NICE: National Institute for Health and Clinical Excellence
NMB: Net monetary benefit
PA: Physical activity
PB: Print-delivered basic intervention condition
PE: Print-delivered environmental intervention condition
QALY: Quality adjusted life years
SD: Standard deviation
SQUASH: Short questionnaire to assess health enhancing physical activity
WB: Web-based basic intervention condition
WE: Web-based environmental intervention condition
WTP: Willingness to pay
