# Supplementary material for: Cost-effectiveness and cost-utility of a Web-based or print-delivered tailored intervention to promote physical activity among adults aged over fifty: an economic evaluation of the Active Plus intervention
Source: Int J Behav Nutr Phys Act. 2014 Sep 28;11:122. doi: 10.1186/s12966-014-0122-z (PMC4189727; doi:10.1186/s12966-014-0122-z)
Supplement: Additional file 1: — Specification of intervention costs. This file gives a detailed specification of the intervention costs. [file 12966_2014_122_MOESM1_ESM.docx]

## Additional file 1. Specification of intervention costs

The intervention costs of the Active Plus intervention are specified in detail in Table 1 in this appendix and can be subdivided in costs per participant (variable costs) and fixed costs. The per participant costs consist of invitation costs, printing and postage costs, printing costs of additional environmental materials and personnel costs, and are explained in detail below.

Invitation costs were registered during the Active Plus trial and consist of all costs incurred to include one participant in the intervention: printing the invitation letters, envelopes, the first questionnaire, postage costs, time required to make information packages ready and sending reminders. Response rates are incorporated in the invitation costs (e.g., to include one participant, an information package has to be send out to at least four potential participants).

The length of the research questionnaire was considerable and may have influenced the response rates. However, in a real-life setting, the length of the questionnaire can be limited (i.e. by only including the questions relevant for generating the tailored advice and excluding the additional questions for research purposes), presumably resulting in increased response rates and lower recruitment costs, printing and postage costs per participant. Based on the literature, we estimate that response rates may increase from 19% in a research-setting [[1](#_ENREF_1)] to 28% in a real-life setting for the printed intervention (considering odds ratio (OR) of 1.48 for shortening the questionnaire [[2](#_ENREF_2)]) and from 12% to 21% for the Web-based intervention (considering an OR of 1.73 [[3](#_ENREF_3)]). Intervention costs were calculated based on these estimated response rates in a real-life setting.

Printing and postage costs include costs to print and mail advice letters to participants (e.g., printed advice letters, envelopes, postage). Specific intervention materials were developed for the intervention condition with environmental information. For the printed intervention, these materials should be printed and thus then result in extra costs.

Personnel costs include the time needed to enter questionnaires (which are part of the intervention) into the tailoring software and preparing questionnaires and advice letters for dispatch. Staff time was valued against the wage of an academic research assistant (€22.29 per hour).

Fixed costs consist of costs for developing environmental materials, since this has to be done for every new region participating in the environment. These costs differ between the printed intervention condition and the Web-based condition because materials are implemented slightly different in the Web-based condition, which required more time. Other fixed costs are costs for tailoring software and hosting costs. Hosting costs for the printed intervention conditions consist of hosting of the tailoring software (e.g., database of advice texts, pictures). For the Web-based intervention conditions, additional hosting costs were incurred for hosting the website in which the intervention was implemented.

***Table 1.*** *Detailed specification of the Active Plus intervention costs (all costs in Euro’s)*

|  | **PB**  *(N = 439)* | **PE**  *(N = 435)* | **WB**  *(N = 423)* | **WE**  *(N = 432)* |
| --- | --- | --- | --- | --- |
| **Cost specification** | | | | |
| Per participant costs |  |  |  |  |
| Invitation costs (including reminders) | 7.87 | 7.87 | 7.54 | 7.54 |
| Printing and postage costs | 6.45 | 7.46 | - | - |
| Printing costs additional environmental materials | - | 1.81 | - | - |
| Personnel costs^1^ | 3.92 | 4.28 | - | - |
| Fixed costs |  |  |  |  |
| Developing environmental materials^2^ | - | 964.97 | - | 1,458.14 |
| Tailoring software | 3,000 | 3,000 | 3,000 | 3,000 |
| Hosting costs | 300 | 300 | 420 | 420 |
| **Total costs** | | | | |
| Total intervention costs^3^ | 11,309.83 | 13,578.59 | 6,610.65 | 8,133.68 |
| Total costs per participant^4^ | 25.77 | 31.21 | 15.53 | 18.83 |

^1^ Based on hourly wage academic research assistant

^2^ Necessary for each participating region

^3^ Total intervention costs = per participant costs * N + fixed costs

^4^ Total cost per participant = total intervention costs / N

As can be seen in Table 1, not all of these costs are applicable to every intervention condition. For the Web-based interventions for example, per participant costs only consist of recruitment costs, because after recruitment, the whole intervention is automated and delivered through the Internet. As a result, every intervention condition has different total costs per participant, with the Web-based basic intervention being the cheapest and the printed intervention with additional environment intervention being the most expensive.

***References***

1. Peels DA, Bolman C, Golsteijn RHJ, De Vries H, Mudde AN, Van Stralen MM, Lechner L: **Differences in Reach and Attrition between Web-based or Print-delivered Tailored Interventions among Adults aged over Fifty.** *J Med Internet Res* 2012, **14:**e179.

2. Sahlqvist S, Song Y, Bull F, Adams E, Preston J, Ogilvie D: **Effect of questionnaire length, personalisation and reminder type on response rate to a complex postal survey: randomised controlled trial.** *BMC Med Res Methodol* 2011, **11:**62.

3. Edwards PJ, Roberts I, Clarke MJ, DiGuiseppi C, Wentz R, Kwan I, Cooper R, Felix LM, Pratap S: **Methods to increase response to postal and electronic questionnaires (Review).** *Cochrane Database Syst Rev* 2009, **3**.
